# Social Isolation in Older Women during the COVID-19 Pandemic: The impact on Quality of Life and Mental health

**DOI:** 10.1093/geroni/igab046.2751

**Published:** 2021-12-17

**Authors:** Victoria Marshall, Robina Sandhu, Kathryn Kanzler, Sara Espinoza, Pamela Keel, Andrea LaCroix, Nicolas Musi, Lisa Kilpela

**Affiliations:** 1 University of Texas Health Science Center San Antonio, San Antonio, Texas, United States; 2 University of Texas Health Science Center, San Antonio, Texas, United States; 3 UT Health San Antonio, San Antonio, Texas, United States; 4 Florida State University, Tallahassee, Florida, United States; 5 University of California at San Diego, San Diego, California, United States

## Abstract

To mitigate the spread of COVID-19, countries worldwide enacted quarantines, particularly for older adults, as mortality from COVID-19 is inequitably distributed among this group. Notably, social isolation in older adults is associated with a heightened risk of cardiovascular, autoimmune, and mental health problems (e.g., depression, anxiety). Furthermore, the mental health of women in particular has been greatly impacted by the pandemic. Although previous research indicates that social isolation among older adults is a “serious public health concern”, less is known about the extent to which the COVID-19 pandemic has exacerbated this issue. The primary objective is to investigate the effects of social isolation on mental health indices and health-related quality of life (HRQOL) in older women in the context of the COVID-19 pandemic. Participants include 77 postmenopausal women (aged 60+) who completed self-report measures online during the COVID-19 pandemic. Controlling for education and annual household income in all analyses, we used linear regression models to investigate the effects of social isolation on depression, anxiety, alcohol use, binge eating, and the 8 domains of the SF-36. Results indicate that, when controlling for education and income, social isolation significantly predicted depression, binge eating, and poorer HRQOL in all 8 domains of the SF-36 (all p’s < .01) Social isolation did not predict anxiety and alcohol consumption when controlling for these sociodemographic variables. Enrollment is ongoing; this poster will report updated results. Results indicate the continued need for creative avenues to improve social connectedness during the COVID-19 pandemic.

